# Treacher Collins Syndrome: Genetics, Clinical Features and Management

**DOI:** 10.3390/genes12091392

**Published:** 2021-09-09

**Authors:** Bożena Anna Marszałek-Kruk, Piotr Wójcicki, Krzysztof Dowgierd, Robert Śmigiel

**Affiliations:** 1 Department of Genetics, Wroclaw University of Environmental and Life Sciences, 51-631 Wroclaw, Poland; 2 Department of Plastic Surgery, Wroclaw Medical University, 50-367 Wroclaw, Poland; p.wojcicki@chirurgiaplastyczna.biz.pl; 3 Head and Neck Surgery Clinic for Children and Young Adults, Department of Clinical Pediatrics, University of Warmia and Mazury, 10-561 Olsztyn, Poland; krzysztofdowgierd@gmail.com; 4 Department of Pediatrics, Division Pediatric Propedeutics and Rare Disorders, Wroclaw Medical University, 51-618 Wroclaw, Poland; robert.smigiel@umed.wroc.pl

**Keywords:** Treacher Collins syndrome, mandibulofacial dysostosis, phenotype, diagnosis, *TCOF1* gene, treacle protein

## Abstract

Treacher Collins syndrome (TCS) is associated with abnormal differentiation of the first and second pharyngeal arches, occurring during fetal development. Features of TCS include microtia with conductive hearing loss, slanting palpebral fissures with possibly coloboma of the lateral part of lower eyelids, midface hypoplasia, micrognathia as well as sporadically cleft palate and choanal atresia or stenosis. TCS occurs in the general population at a frequency of 1 in 50,000 live births. Four subtypes of Treacher Collins syndrome exist. TCS can be caused by pathogenic variants in the *TCOF1*, *POLR1D*, *POLR1C* and *POLR1B* genes. Genetically, the *TCOF1* gene contains 27 exons which encodes the Treacle protein. In *TCOF1*, over 200 pathogenic variants have been identified, of which most are deletions leading to a frame-shift, that result in the formation of a termination codon. In the presented article, we review the genetics and phenotype of TCS as well as the management and surgical procedures utilized for treatment.

## 1. Genetics

Treacher Collins syndrome (TCS, OMIM 154500), also referred to as Franceschetti syndrome or mandibulofacial dysostosis (MFD1), is a rare developmental disorder. The disorder has been described by Berry [1], Treacher Collins [2] and Franceschetti and Klein [3]. The incidence is about 1 in 50,000 live births [4] with 40% of cases involving a family history, while the remaining 60% occur as a result of de novo mutations.

TCS syndrome is genetically heterogeneous [5]. The literature describes four clinical subtypes: Treacher Collins syndromes 1: TCS1 is caused by pathogenic variants of the *TCOF1* gene; Treacher Collins syndromes 2: TCS2 is caused by pathogenic variants in the *POLR1D* gene; and Treacher Collins syndromes 3: TCS3 caused by pathogenic variants in the *POLR1C* gene and lately identified *POLR1B* gene as a new gene responsible for a novel Treacher Collins syndromes 4: TCS4 [6] (Table 1).

No association has been identified between the clinical features of patients and the gene in which the alterations occur.

The *TCOF1* gene is encoded by 27 exons. Two exons discovered latest are: exon 6A of length 231bp localized between exons 6 and 7 and exon 16A, 108bp in length, localized between exons 16 and 17 [11]. *TCOF1* encodes a 144 kDa nuclear phosphoprotein called Treacle which consists of 1411aa [12], while the transcript containing the additional exon 6A encodes a protein longer by 77aa [11].

In the central region of treacle are the multiple casein kinase 2 (CK2)/protein kinase C (PKC) phosphorylation sites [13]. Treacle contains a putative nuclear export signal at the N-terminus and nuclear import signals at the C-terminus, suggesting dynamic localization of treacle [14]. A LisH motif is located at the N-terminus of treacle [15].

Treacle is involved in the transport of proteins and ribosomal subunits between the nucleolus and cytoplasm [16]. This protein supports ribosomal DNA gene transcription by interacting with the UBF (upstream binding factor) [17]. Treacle is involved in ribosome biogenesis by binding and requiring Pol I, UBF, Nopp140 to the rDNA promoter. UBF is usually tightly bound by treacle. A decrease in treacle could cause UBF rapid displacement from rDNA and then to the inhibition of rRNA synthesis. Increased expression of treacle may interfere with cisplatin-induced apoptosis [18]. Thus, both silencing and ectopic expression of *TCOF1* coding treacle can cause apoptosis of neural crest cells during embryogenesis by influence on crucial apoptotic regulators [14].

Loss of treacle protein function in neuroblastoma cells affects the expression of genes that are involved in proliferation, apoptosis and cell cycle.

Ciccia et al. [19] identified treacle as a potential component of the NBS1 protein complex. Subunits of RNA polymerase I (POLR1A, B and E) were found to be components of *TCOF1* complexes. They have also discovered *TCOF1* as a DDR (DNA damage response) factor, that recruits NBS1 to nucleoli after DNA damage. DNA damage due to oxidative stress occurs during embryonic development [15].

**Figure 1:**
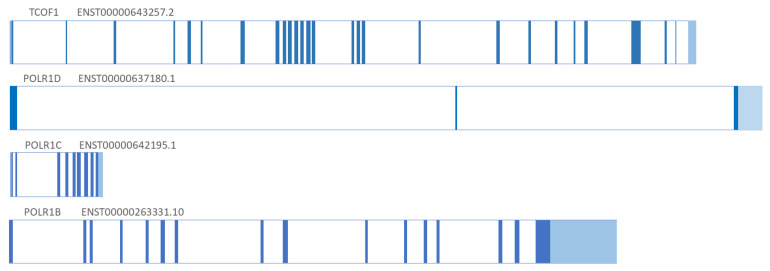
Genetic structure of genes responsible for TCS. Ref. [20,21].

Dauwerse et al. discovered that *POLR1C* and *POLR1D* genes are involved in TCS syndrome by detecting deletion and pathogenic variants in the *POLR1D* and *POLR1C* genes in patients with TCS [7]. The *POLR1C* and *POLR1D* genes were discovered more than 10 years after the discovery of the *TCOF1* gene. The *POLR1D* gene is encoded by 3 exons, *POLR1C* by 9 exons, while the *POLR1B* gene consists of 15 exons. The structure of TCS-associated genes, their exons, and differences in length are presented (Figure 1).

Pathogenic variants in *POLR1D* are autosomal dominant and autosomal recessive, in *POLR1C* are autosomal recessive, while in *TCOF1* and *POLR1B* genes are autosomal dominant. Variants in *POLR1D* lead to haploinsufficiency of gene, whereas in *POLR1C* lead to functional depletion of the gene [7].

*POLR1C* and *POLR1D* are subunits that are shared between RNA polymerases I and III. RNA polymerase I and III are involved in ribosomal RNA transcription. Insufficient amounts of Pol I or Pol III can lead to a reduced number of mature ribosomes in neural crest cells during embryonic development, which could affect apoptosis of proper initially formed first and second branchial arches causing craniofacial abnormalities [7].

The *POLR1B* gene is a unique subunit of RNA polymerase I. Pathogenic variants of the *POLR1B* gene can lead to p53-dependent apoptosis and consequent cranioskeletal defects [6].

Over 200 mutations have been reported in the *TCOF1* gene including deletions, insertions, substitutions [15,22,23,24,25,26,27]. Exons 10, 15, 16, 23 and 24 are hotspots in *TCOF1*. Conte et al. [15] detected 15 different *TCOF1* pathogenic variants in multiple exons: 3, 5, 6, 10, 12, 13, 15, 16, 18, 20, 22, 23, 24. The effect of most *TCOF1* pathogenic variants is haploinsufficiency.

In most TCS causes, the pathogenic variants are small deletions from 1 to 40 nucleotides, that result in premature termination of codons, producing a truncated protein [8]. Chen et al. [28] detected about 63% deletions ranging in size from 1–38 nucleotides in Chinese patients with TCS.

Gross deletions of *TCOF1* were identified with a 5% frequency in those with TCS [29]. Beygo et al. [30] identified a 3.367kb deletion in one patient with a clinical diagnosis of TCS. This deletion concerned exon 3 and is the first described single exon deletion within the *TCOF1* gene. Liu et al. [31] identified a novel 2–6 exon deletion of *TCOF1*. This was the first report of a fetal for TCS in a Chinese population.

Kantaputra et al. [32] discovered a novel *TCOF1* pathogenic variant, c.4138_4142del, p.Lys1380GlufsTer12 in patients with TCS, who did not have the typical TCS phenotype. In these cases were observed short cranial bases, hypoplastic maxilla and mandible. The next most common pathogenic variants of the *TCOF1* gene are insertions. The longest insertion, c.484_668ins185bp, was localized to exon 5 in twin sisters [33]. Zhang et al. [27] identified five novel variants (two nonsense, one missense and one splicing) in *TCOF1* in Chinese patients. Zeng et al. [26] reported a nonsense pathogenic variant (c.1622G > A) in exon 11 of *TCOF1*.

Dauwerse et al. [7] detected pathogenic variants in genes *POLR1C* and in *POLR1D* (20 heterozygous variants) in TCS patients, while a report by Sanchez et al. [6] identified three novel pathogenic variants in *POLR1B*.

In the case of Treacher Collins syndrome patients, no correlation is observed between the type of the pathogenic variants and the phenotype.

## 2. Clinical Features–Phenotype

Symptoms of TCS are clinically very variable and include following mean malformations/features:

- external and middle ear abnormalities including microtia with conductive hearing loss attributed most commonly to malformation of the ossicles,
- bilateral and symmetric downslanting palpebral fissures,
- coloboma or notching of the lateral part of lower eyelids with medial absence or sparse of the eyelashes and tear ducts defect,
- hypoplasia of the facial bones with micrognathia and retrognathia as well as zygomatic bones. Hypoplasia of the zygomatic bones and mandible can cause significant feeding and respiratory difficulties.

Other common manifestations include cleft palate, unilateral or bilateral choanal stenosis or atresia and pharyngeal hypoplasia. Most patients with TCS have unaffected intellect, and TCS occurs in both genders equally (Table 2).

**Figure 2:**
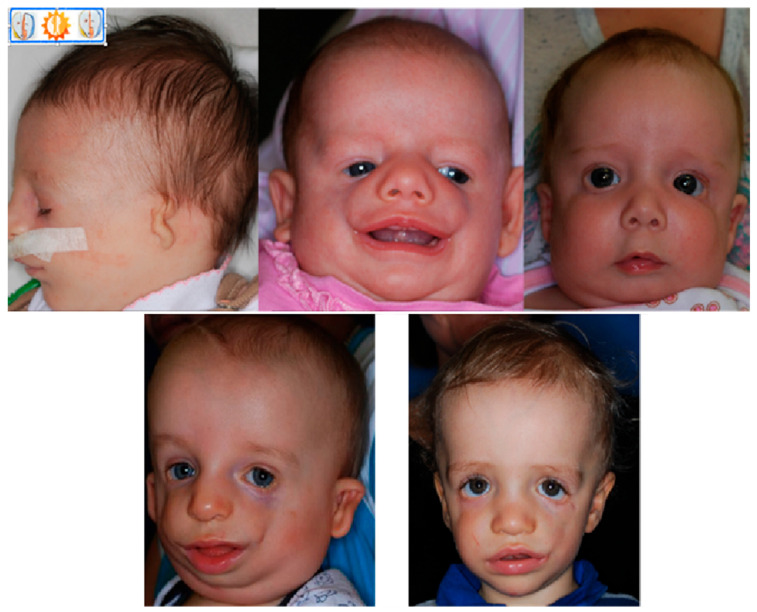
Patients with TCS at different ages, visible variations in defect severity.

The phenotypic features of TCS are observed bilaterally, and disorder intensity is determined by skeletal deformities. The lateral and lower orbital walls are shorter due to hypoplasia of the zygomatic bone, there is narrowing and flattening of the bony face, as well as displacement of the outer corner of the eye and symmetric downslanting palpebral fissures [36]. A forward protruding nose is often observed, as well as abnormal occlusion, and in more severe cases the falling back of the tongue resulting in breathing problems. The severity of the deformities does not increase with age (Figure 2). Patients with TCS have various degrees of deformed auricles, leading to partial or complete deafness. Most TCS patients have unilateral or bilateral conductive type hearing loss. Conductive hearing impairment can be attributed mainly to malformations of the middle ear, which are similar in patients with hearing defects or lack of auditory ossicles.

In 60% of children is noticed a poor quality of speech. Speech difficulties have resulted from hearing loss problems and speech acquisition and are associated with structural defects of the ears. Researchers noted that speech therapy may be more effective after structural normalization; however, it is also recommended before such normalization. Patients with TCS may use speech through expressions such as gestures, facial expressions, and vocalizations, which the speech pathologist should consider to contribute to social interactions. Children who have tracheostomies require special attention from speech pathologists.

Clinical diagnosis of patients with TCS may be difficult as they possess features similar to other disorders, such as oculoauriculovertebral spectrum (known as Goldenhar syndrome), Miller and Nager syndrome, as well as mandibulofacial dysostosis with microcephaly. These disorders are caused by abnormal development of the first and second branchial arches of embryonic development [25]. In the case of oculoauriculovertebral spectrum disorders, the genes responsible for the disease have not been described in the literature. Nager syndrome is an autosomal dominant disorder. It is caused by variants in the *SF3B4* gene, which is located on chromosome 1q21.2. Patients with Nager syndrome are characterized by the following phenotype: downslanting palpebral fissures, hypoplasia of zygomatic bone, micrognathia, cleft lower eyelid, dysplastic ears associated with hearing loss, combined with predominantly radial limb defects such as lateral absence or hypoplastic of thumbs and hypoplasia of the radius with radioulnar synostosis. Miller syndrome is an autosomal recessive disorder caused by mutations in the *DHODH* gene, which is located on chromosome 16q22.2. Patients with Miller syndrome are characterized by malar hypoplasia, micrognathia, microstomia and a cleft lip and/or palate. The limb defects in Miller syndrome are distinctive, consisting of an absence or incomplete development of the 5th digital ray of all four limbs, and, frequently, forearm abnormalities (the ulna and radius can be short with occasional radio-ulnar synostosis). Pathogenic variants in the *EFTUD2* gene are responsible for mandibulofacial dysostosis with microcephaly (MFDM), characterized by the following phenotypic features: microcephaly, asymmetry of facial features with unusual ears with preauricular tags, sensorineural deafness, a small jaw and major defects including choanal atresia, cleft palate, cardiac defects and even esophageal atresia as well as significant developmental delay and speech delay.

## 3. Management and Surgical Treatment

**Figure 3:**
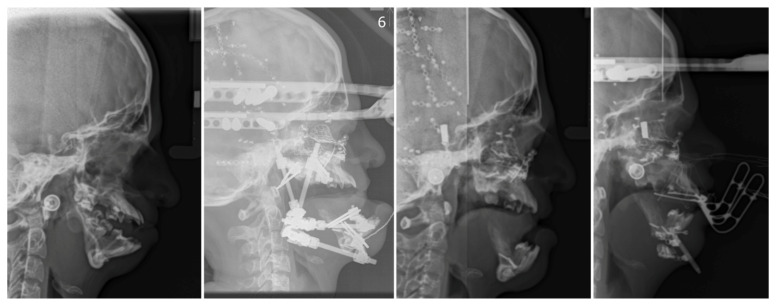
Cephalometric radiographs showing the basic steps of treatment related to the process of mandibular and maxillary osteodistraction aimed at improving facial contour and opening the upper airway.

Patients with TCS need specialized, multidisciplinary treatment involving pediatrics, clinical genetics, otolaryngologist, orthodontist, audiologist, psychologist and having major impact variable types of surgeons. Of significant importance is (computed tomography) CT scanning, used to document the anatomy of the head in order to prepare a plan for surgery, to recognize defects of hearing organs as well as to document progress in different stages of treatment (Figure 3). Neuro-ophthalmologic evaluation of children is necessary to determine extraocular muscle function and to assess corneal exposure and visual acuity due to orbital defects. An echocardiogram is used to identify heart defects and also ophthalmologic and dental anomalies assessments are made.

Treatment can be divided into three main phases depending on the age of the patient.

**Figure 4:**
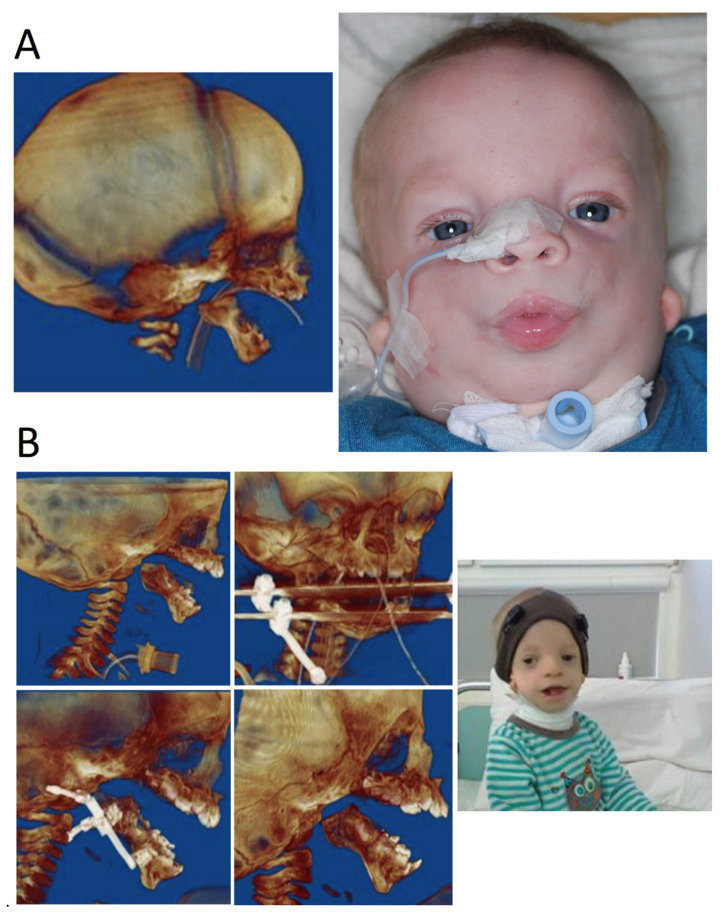
The same patient at different ages during different stages of treatment. (**A**) Infant with TCS, CT image of 3D reconstruction, patient with tracheostomy. (**B**) Few years-old patient. 3D images of reconstruction during treatment—multistage mandibular osteodistraction; patient after completion of osteodistraction process, removed tracheostomy tube.

**Figure 5:**
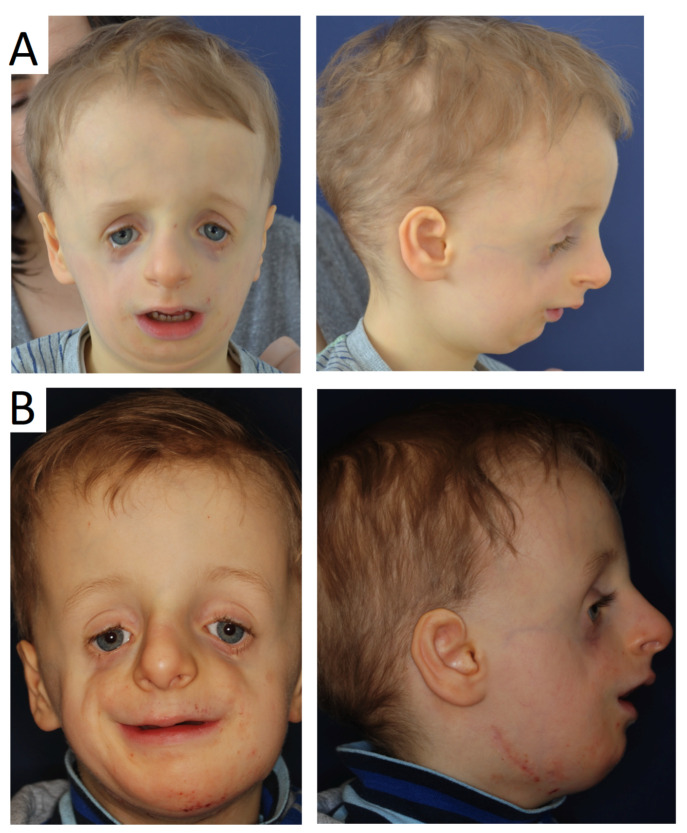
Patient at the age of 4 before (**A**) and after (**B**) mandibular distraction prior to further stages of treatment.

**Figure 6:**
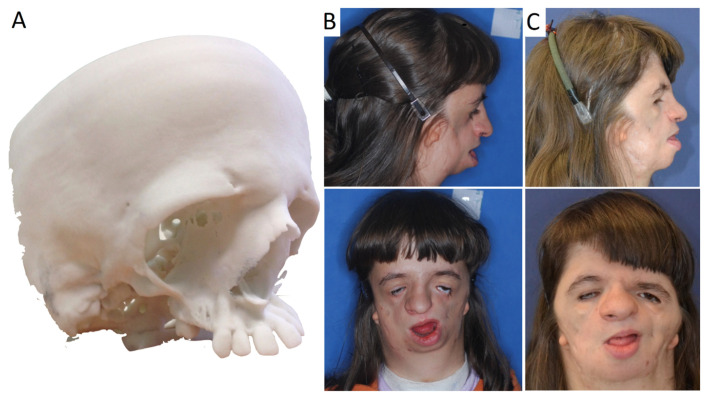
Teenage female patient with TCS. (**A**) Stereolithographic model with visible bone deformations. (**B**) Side and frontal view before major treatment, large deformation of the middle face and mandible resulting in closure of the upper airway, absence of zygomatic bone, coloboma of the lower eyelids. (**C**) Patient after multistage reconstructive treatment forming the shape of face, transplant taken from the cranial vault mounted in place of the zygomatic bone; despite late notification of the therapy, noticeable improvements in facial features.

**Figure 7:**
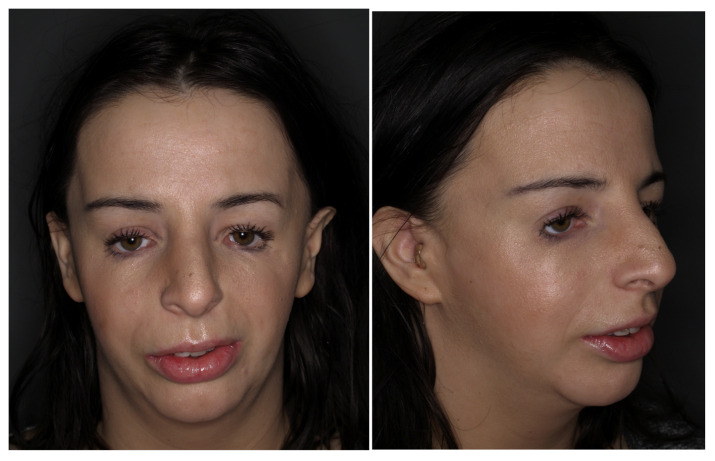
Adult patient with TCS after correction treatment.

- Birth to age 2 years—facilitation of elementary living functions: breathing, e.g., tracheostomy (Figure 4), feeding and hearing as well as vision and heart.
- Age 3 to 12 years—speech therapy, integration into society, bone reconstructions including mandibular (Figure 5), which can prevent progression of defects as well as ophthalmological and orthodontic support. Pre-school children usually receive the same treatment.
- Age 13 to 18—orthognathic therapies, maxillomandibular and nasal reconstruction as well as integration into society. Multistage reconstructive treatment forming face is possible in this age and makes noticeable improvements (Figure 6). However, if possible, it is recommended to start therapy at an earlier age. The sooner therapy begins gives the higher possibility of achieving optimal results (Figure 7).

Treatment must be started as early as the first year of life in cases where there is a life-threatening risk, e.g., nocturnal apnea in children with underdevelopment of the mandible and hypertrophy of the tongue. In the most severe cases, this can lead to suffocation (apnea). In these cases, a mandibular distraction is most effective. The correction of macrostomia or cleft lip surgery is generally performed in infancy. Similarly, due to the need to protect the eyeball, coloboma surgery is generally carried out in the first year of life (even in the first months) if indicated.

To determine the severity of mandibular underdevelopment, the Kaban classification is used (types I–III). For patients with TCS, surgical treatment is critical and often begins in the first year of life and continues until growth is complete. The shortening of the mandible and falling back of the tongue results in upper airway obstruction. If macrostomia is present, surgery is needed before the child is one year of age. The lip is sewn at the age of 6 months and the cleft palate between the first and second years of life [37].

The cleft upper eyelid (coloboma) should be operated on before the end of the first year of life. This is necessary to create mechanical eye protection and to ensure proper corneal hydration. Misalignment of the eyelid crevices is corrected by lateral canthopexy, and eyelid defects and coloboma are corrected with local plasty eyelid flaps.

Another phenotypic feature seen in children with TCS is symmetric downslanting palpebral fissures, surgery for which begins at preschool age. The procedure involves displacement of a long dermal-musculocutaneous flap from the upper eyelid to the lower eyelid and canthopexy [38]. In cases of complete auricular microtia, reconstruction is performed with autogenous cartilage. Underdevelopment of the mandible is treated by conducting bone distractions, with more severe cases requiring treatment between 6 and 10 years of age [39].

**Figure 8:**
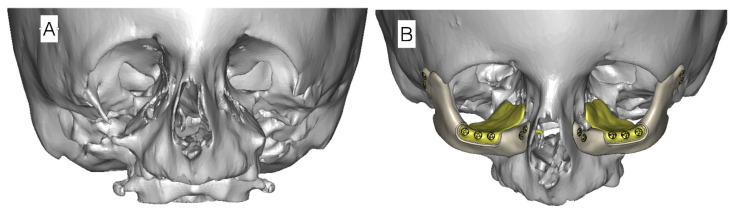
Example of virtual planning of zygomatic bone reconstruction with alloplastic implants. (**A**) Computed tomography image prior to transplantation in a patient with missing zygomatic bone. (**B**) CT scan image with planned alloplastic zygomatic bone reconstruction and individual orbital floor recontouring plates.

Orbital cavity wall deformations are operated simultaneously with proper eyelid positioning. To improve facial appearance due to wide and long nose structure, horizontal chin osteotomy and rhinoplasty can be performed [40]. Orbital floor defects are reconstructed with allogenic or individual orbital floor implants (Figure 8). Reconstruction of zygomatic and orbital bone deficiencies should not be performed before the age of 5 years unless there are severe problems with corneal exposure.

## 4. Conclusions

Molecular diagnosis plays a very significant role for patients with Treacher Collins syndrome in the prenatal and postnatal periods. Genetic counselling is very important for every family with a TCS child. Each patient with TCS is unique and treatment should be developed by a multidisciplinary team. As there are no clinical differences between the four subtypes of Treacher Collins syndrome, the surgical treatment also does not differ according to the type of pathogenic variants in the genes described. Apart from age, it is the appearance, location and severity of the deformity that determines treatment priorities.

The complete etiology of TCS is not yet fully understood. In about 10% of cases, it remains unknown what implies the disorder. This may indicate a role of other genes or other genetic mechanisms in the pathogenesis of TCS [28].

## Figures and Tables

**Table 1:** Classification of genes and subtypes of Treacher Collins syndrome (TCS) (ORPHA: 861).

Phenotype		Gene						
TCS Subtype	OMIM	Name	OMIM	Inheritance	ID	Chromosome Locus	Frequency of TCS Pathogenic Variant [6,7,8]	Product of Gene
TCS1	154500	*TCOF1*	606847	AD	6949	5q32-q33	86%	Treacle protein
TCS2	613717	*POLR1D*	613715	AD, AR	51082	13q12.2	6%	DNA-directed RNA polymerases I and III subunit RPAC2
TCS3	248390	*POLR1C*	610060	AR	9533	6p21.1	1.2%	DNA-directed RNA polymerases I and III subunit RPAC1
TCS4	618939	*POLR1B*	602000	AD	84172	2q14.1	1.3%	DNA-directed RNA polymerase I subunit RPA2

AD—autosomal dominant; AR—autosomal recessive; OMIM—Online Mendelian Inheritance in Man. Ref. [9,10].

**Table 2:** Classic features of Treacher Collins syndrome.

Classic Feature	Symptom, Feature	Occurrence in Affected Individuals
Very frequent	Downslanting palpebral fissures	89–100%
	Malar hypoplasia/hypoplasia of zygomatic complex	81–97%
	Conductive hearing loss	83–92%
	Mandibular hypoplasia/micrognathia	78–91%
Frequent	Atresia of external ear canal	68–71%
	Microtia	10–77%
	Coloboma (notching) of the lower lid	54–69%
	Delayed speech development	57–63%
	Asymmetry	52%
	Preauricular hair displacement	24–49%
Rare	Nasogastric tube or gastrostomy in neonates	28%
	Cleft palate	21–33%
	Intubation or tracheostomy in neonates	12–18%
	Choanal stenosis/atresia	13–25%
	Cardiac malformation	11%
Very rare	Rachis malformation	7%
	Renal malformation	4%
	Microcephaly	3%
	Intellectual disability Delayed motor development	1.7–10%
	Limb anomaly	1.5%

Ref. Vincent et al. [8], Teber et al. [34], Splendore et al. [35].

## Data Availability

This is a review article. The study did not report any data.
