# Correction: Marszałek-Kruk et al. Treacher Collins Syndrome: Genetics, Clinical Features and Management. *Genes* 2021, *12*, 1392

**DOI:** 10.3390/genes16111391

**Published:** 2025-11-20

**Authors:** Bożena Anna Marszałek-Kruk, Piotr Wójcicki, Krzysztof Dowgierd, Robert Śmigiel

**Affiliations:** 1 Department of Genetics, Wroclaw University of Environmental and Life Sciences, 51-631 Wroclaw, Poland; 2 Department of Plastic Surgery, Wroclaw Medical University, 50-367 Wroclaw, Poland; 3 Head and Neck Surgery Clinic for Children and Young Adults, Department of Clinical Pediatrics, University of Warmia and Mazury, 10-561 Olsztyn, Poland; krzysztofdowgierd@gmail.com; 4 Department of Pediatrics, Division Pediatric Propedeutics and Rare Disorders, Wroclaw Medical University, 51-618 Wroclaw, Poland

**Error in Figure**

In the original publication [1], there was a mistake in Figure 2 as published. Patient whose image was included in the image, has withdrawn their approval according to their rights granted by GDPR. The corrected Figure 2 appears below. The authors state that the scientific conclusions are unaffected. This correction was approved by the Academic Editor. The original publication has also been updated.

## Figures and Tables

**Figure 2:**
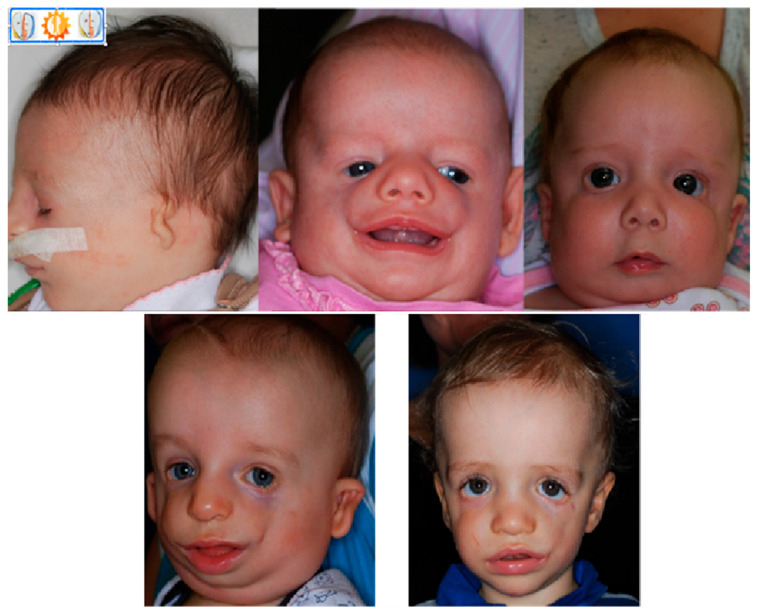
Patients with TCS at different ages, visible variations in defect severity.
